# Efficacy and Safety of Lamivudine or Telbivudine in Preventing Mother-to-Child Transmission of Hepatitis B Virus: A Real-World Study

**DOI:** 10.1155/2020/1374276

**Published:** 2020-04-27

**Authors:** Zhenhua Li, Xuefei Duan, Yuhong Hu, Mingfang Zhou, Min Liu, Kai Kang, Haodong Cai, Wei Yi, Dong Fu, Xuesong Gao

**Affiliations:** ^1^Department of Obstetrics and Gynecology, Beijing Ditan Hospital, Capital Medical University, Beijing, China; ^2^Department of General Medicine, Beijing Ditan Hospital, Capital Medical University, Beijing, China; ^3^Hepatology Clinic, Beijing Ditan Hospital, Capital Medical University, Beijing, China

## Abstract

**Background:**

There are few large sample studies evaluating the safety and efficacy of lamivudine (LAM) or telbivudine (LdT) in preventing hepatitis B mother-to-child transmission (MTCT) in highly viremic mothers in the third trimester of pregnancy in real-world settings. The purpose of this study was to analyze a large sample size of HBV-infected mothers to better understand the safety and efficacy of LAM and LdT under the aforementioned criteria.

**Methods:**

During the period of November 2008 to November 2017, we retrospectively enrolled mothers with HBV DNA > 1 × 10^6^ IU/mL who received LAM or LdT during the third trimester of pregnancy and compared them to untreated mothers. All mothers were divided into the three following groups: the LAM group, the LdT group, and the control group.

**Results:**

A total of 2624 HBV-infected mothers were enrolled in the study, with 363 in the LAM group, 1283 in the LdT group, and 978 in the control group. The MTCT rates were significantly lower in the LAM or LdT group than that in the control group (0.4% or 0.3% versus 9.0%, *P* < 0.001). Infants born to untreated mothers had a significantly higher risk of HBV infection (OR = 28.6, 95% CI: 10.4–78.7, *P* < 0.001). There were no significant differences in perinatal complications between the three groups (*P* > 0.05). There were also no differences for gestational age or infants' height, weight, Apgar scores, or birth defect rates. Postpartum discontinuation of antiviral therapy did not seem to increase the risk of postpartum alanine aminotransferase (ALT) flare.

**Conclusion:**

LAM or LdT treatment initiated in the third trimester for mothers with HBV DNA > 1 × 10^6^ IU/mL was equally safe and effective in preventing MTCT.

## 1. Introduction

Hepatitis B virus (HBV) severely threatens the health of an estimated 257 million people worldwide, according to the “Global Hepatitis Report” released by WHO in 2017. In 2015, approximately 887,000 people died from complications from hepatitis B, including cirrhosis and liver cancer [1]. Approximately 93 million people in China are chronically infected with HBV [2]. Vertical transmission is the main route of HBV transmission in China, accounting for 35%–50% of HBV infections [3]. Although the combination of hepatitis B vaccine and hepatitis B immunoglobulin (HBIG) immunization reduces the rate of mother-to-child transmission (MTCT), immunoprophylaxis fails in 5%–10% of infants born to mothers with high HBV DNA levels [4, 5].

A growing number of studies indicate that the use of lamivudine (LAM), telbivudine (LdT), or tenofovir in the third trimester of pregnancy can reduce MTCT [6–8]. Women with HBV DNA > 200,000 IU/mL in the third trimester are recommended antiviral treatment to prevent MTCT [2, 9, 10]. However, there are no real-world studies based on large sample sizes that study the use of antiviral therapy in late pregnancy for preventing MTCT. We conducted a retrospective study with a sample size of over 2000 pregnant mothers with HBV DNA levels > 1 × 10^6^ IU/mL to assess the safety and efficacy of the third-trimester use of LdT or LAM versus no treatment and monitored the safety for mothers and infants during the postpartum period.

## 2. Methods

### 2.1. Patients

The retrospective cohort study recruited HBsAg-positive pregnant women at a routine antenatal screening at the Department of Gynecology and Obstetrics in Beijing Ditan Hospital, Capital Medical University from November 2008 to November 2017. Patients were diagnosed with chronic HBV infection (CHB) according to the Guideline of Prevention and Treatment for Chronic Hepatitis B (2015 Update) [2].

All pregnant women were enrolled at 28 ± 4 weeks of gestation. The eligible criteria included the following: (1) HBsAg and (2) HBV DNA levels > 1 × 10^6^ IU/mL at 28 ± 4 weeks of gestation. The exclusion criteria were as follows: (1) major systemic disease, including renal insufficiency, heart disease, malignant neoplasm, liver cirrhosis, or hepatic decompensation; (2) history of amniocentesis during pregnancy; (3) a familial history of genetic disease in either member of a couple; (4) coinfection with human immunodeficiency virus, hepatitis C virus, hepatitis D virus, toxoplasmosis, syphilis, rubella, or cytomegalovirus; (5) treatment with HBIG during pregnancy; (6) history of two or more spontaneous abortions; (7) previous delivery of a child with a deformity; and (8) infants who did not complete HBIG or 3-dose HBV vaccine.

Pregnant women were divided into the LAM group, the LdT group, and the control group based on individual preference after the benefits and risks of initiating antiviral therapy in early pregnancy were explained to them. Antiviral drug selection was at the patient's discretion. Pregnant women who received LAM treatment at 28 ± 4 weeks of gestation were classified into the LAM group. Pregnant women in the LdT group initiated LdT treatment also at 28 ± 4 weeks of gestation. The control group consisted of pregnant women with CHB who declined antiviral therapy. All participants signed a consent form.

All infants received 100–200 IU of HBIG at birth. They received 10 *μ*g of hepatitis B vaccine at birth, 1 month, and 6 months. This study was approved by the institutional review board of Beijing Ditan Hospital and informed consent was waived.

### 2.2. Data Collections

Maternal data and pediatric data were retrospectively reviewed and collected using an electronic medical record system of Beijing Ditan Hospital. Maternal data included age, parity, serum alanine transaminase (ALT) levels, creatine kinase (CK), serum creatinine (Cr), HBV serological markers, HBV DNA levels, presence or absence of antiviral therapy, and pregnancy complications. Infants' data included gestational weeks, delivery mode, infant sex, birth weight, birth length, Apgar score (1 min) at birth, infant adverse events, birth defect, HBV serological markers, and HBV DNA viral loads at birth and at 28 weeks.

### 2.3. Outcome Assessment

The primary outcome was the MTCT rate. The MTCT rates of infants born to mothers with LAM or LdT treatment were compared with those of infants born to untreated mothers. The MTCT rate was defined by HBsAg seropositivity or HBV DNA positivity at 7–12 months postpartum.

The secondary outcomes were the following: (1) maternal HBV DNA levels before delivery; (2) maternal complications during the prenatal period, including premature rupture of membranes, postpartum hemorrhages, meconium staining of the amniotic fluid, fetal distress, and creatine kinase (CK). Elevations of CK grade 2 were defined as CK ≥ 5 × the upper limit of normal (ULN). (3) Infant outcomes were assessed by gestational age, weight, length, Apgar score, prematurity (<37 weeks), low birth weight (LBW) (<2500 grams), birth defect rates, and maternal liver function at postpartum.

### 2.4. Laboratory Testing

All the laboratory tests were performed in the hospital's central laboratory. HBV serological markers (HBsAg, anti-HBs, HBeAg, anti-HBe, and anti-HBc) were measured using a chemiluminescent microparticle immunoassay (Architect i2000 Analyzer; Abbott Diagnostics, USA). The serum HBV DNA level was quantified using a real-time polymerase chain reaction (PCR) system (LightCycler 480 Real-Time PCR System, Roche Life Science), with a lower limit of 100 IU/mL. ALT (normal 7–40 U/L) and CK (normal 40–200 U/L) levels were tested using a Hitachi 7600 fully automatic biochemical analyzer (Wako Pure Chemical Industries, Ltd., Tokyo, Japan).

### 2.5. Statistical Analysis

All data were analyzed using Stata 11.0. Categorical variables were presented as numbers and percentages and compared by chi-square analysis (Fisher's exact test if needed). Normally distributed continuous variables were expressed as mean ± standard deviation (SD). Serum HBV DNA levels were converted to a common logarithm. Comparisons between multiple groups were performed by using analysis of variance (Bartlett's test). If the analysis of variance test was significant, the *P* value for pairwise comparisons was computed with Student's *t*-test. The pretherapy data were compared with posttherapy by a paired-sample *t*-test. Quantitative data nonnormally distributed were presented as median and minimum-maximum values. The Kruskal-Wallis test was used to compare multiple groups of variables. If the test was significant, the Wilcoxon signed-rank test for pairwise comparison was performed. Logistic regression was used to calculate the risk ratio (OR) and 95% confidence interval (95% CI) of HBV infection in infants in the untreated group. *P* < 0.05 was considered statistically significant.

## 3. Results

### 3.1. Baseline Maternal Characteristics

A total of 2624 HBsAg-positive pregnant women were enrolled, including 363 (13.8%) in the LAM group, 1283 (48.9%) in the LdT group, and 978 (37.3%) in the control group. Their clinical characteristics are shown in Table 1. There were no significant differences in clinical features among the three groups except the ALT and HBV DNA levels (*P* < 0.001).

### 3.2. Efficacy Assessment in Mothers and Infants

At the 7–12-month follow-up visits, 236 (64.5%) infants were born to mothers in the LAM group with 1 (0.4%) case infected with HBV, and 930 (71.6%) infants were born to mothers in the LdT group with 3 (0.3%) cases infected with HBV. Finally, 781 (79.4%) infants were born to mothers in the control group, and 70 (9.0%) cases were infected with HBV. There was a significant difference in the rate of MTCT among the three groups (*P* < 0.001). Furthermore, there was no significant difference in the rate of MTCT between the LAM group and the LdT group (*P* = 1.000). The rate of MTCT was higher in the control group, compared to the rate in the LAM (*P* < 0.001) and LdT (*P* < 0.001) groups. The risk of HBV infection in infants born in the control group was higher than that in infants born to mothers taking LAM or LdT (OR = 28.6, 95% CI: 10.4–78.7, *P* < 0.001).

**Figure 1:**
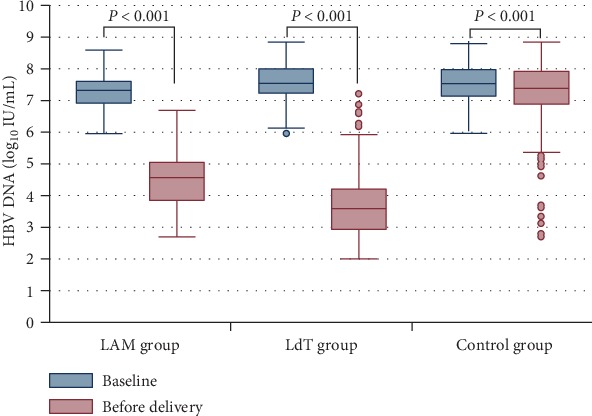
HBV kinetics in pregnant mothers infected with hepatitis B virus in three groups. Comparison of HBV DNA levels between baseline and before delivery of CHB mothers. HBV loads were significantly decreased before delivery among the three groups (*P* < 0.001).

From 28 weeks of gestation to delivery, the HBV DNA levels in the three groups decreased significantly (*P* < 0.001) (Figure 1). The HBV DNA levels in the LAM (4.43 ± 0.92 log_10_ IU/mL) or LdT group (3.68 ± 0.85 log_10_ IU/mL) were significantly lower than those in the control group (7.36 ± 0.85 log_10_ IU/mL) before delivery (*P* < 0.001). In addition, the degree of HBV DNA reduction in the LAM or LdT group was greater compared to the degree of HBV DNA reduction in the control group (*P* < 0.001). The proportion of patients with undetectable HBV DNA in the LAM (9.6% or 35/363) or LdT (18.6% or 239/1283) group was also significantly higher than in the control group (0.5% or 5/978) (*P* < 0.001).

### 3.3. Maternal Safety

The maternal complications during the prenatal period are summarized in Table 2. The maternal complications were similar among the three groups except for the admission rate due to elevations in ALT levels. The admission rate in the control group was significantly higher than that in the LAM or LdT group (*P* = 0.016).

In total, 191 (52.6%) in the LAM group, 1121 (87.4%) in the LdT group, and 238 (24.3%) in the control group were tested for CK levels before delivery. The median of CK levels was 59 U/L (7–411) in the LAM group, 65 U/L (13–1367) in the LdT group, and 54 U/L (15–714) in the control group, indicating a significant difference among the three groups (*χ*^2^ = 28.071, *P* < 0.001). The mothers had a higher frequency of elevation in CK levels in the LdT group than in the LAM group (*z* = −2.830, *P* = 0.005) or in the control group (*z* = 4.277, *P* < 0.001). The CK levels were similar in the LAM and control groups (*z* = 1.400, *P* = 0.162). One mother had a CK grade 2 elevation in the LdT group, while no mothers had grade 2 or higher CK elevation in the LAM group or the control group.

### 3.4. Infant Safety

In the LAM group, 363 women had 366 infants, with three women having twin pregnancies. In the LdT group, 1283 women had 1298 infants with 15 twins, while 978 women in the control group had 984 infants with 6 twins. There were no significant differences in the cesarean rate, gestational age, weight, height, Apgar score, LBW rate, birth defect rate, and death rate during the prenatal period among the three groups (Table 3).

### 3.5. Changes in Maternal ALT Level after Delivery

Three hundred and sixteen (87.1%) mothers in the LAM group and 1065 (83.0%) mothers in the LdT group discontinued antiviral therapy within 3 months after delivery, and the remaining mothers continued treatment due to previous ALT abnormalities. In the control group, 108 (11.0%) mothers started antiviral therapy due to abnormal ALT within 6 months after delivery. ALT elevations ≥ 2 × ULN occurred in 67 (18.5%), 343 (26.7%), and 355 (36.3%) mothers in LAM group, LdT group, and the control group, respectively. There were significant differences among the three groups (*P* < 0.001). Further pairwise comparisons brought out a significantly higher proportion of patients with ALT elevation ≥ 2 × ULN in the control group (*P* < 0.001).

## 4. Discussion

Vertical transmission is a major way of HBV transmission and the main cause of chronic HBV infection. A high viral load is the main cause of immunization failure of HBV [11, 12]. Previous studies have demonstrated that treatment with LAM, LdT, or tenofovir disoproxil (TDF) during the third trimester can effectively reduce the risk of transmission of HBV [13–17]. However, there is still a lack of a real-world study with a large sample size.

Compared to the control group, there was a greater decline of HBV DNA in mothers and a higher proportion of mothers with undetectable HBV DNA before delivery in the LAM and LdT groups. Infants born to mothers in the control group were 28.6 times more likely to be infected with HBV than those born to mothers with LAM or LdT treatment (OR = 28.6, 95% CI: 10.4–78.7, *P* < 0.001), indicating that LAM and LdT effectively reduced the rate of HBV MTCT. Meanwhile, the rate of infection did not differ significantly between the LAM and LdT groups. This result is consistent with a previous study in which a meta-analysis including 9228 mother-infant pairs in 59 studies (32 RCTs and 27 non-RCTs) demonstrated that LAM, LdT, and TDF were equally effective in blocking HBV MTCT [18]. The MTCT rate may be significantly reduced due to decreased HBV DNA levels. Han et al. conducted a meta-analysis evaluating the efficacy of LAM treatment in late pregnancy [6]. A total of 1693 mothers in 15 randomized controlled trials were included in this meta-analysis. The authors demonstrated that the HBV MTCT rate was significantly lower in the LAM group than in the untreated group if maternal HBV DNA levels were decreased to <1 × 10^6^ copies/mL.

Since antiviral therapy was initiated in the third trimester of pregnancy, we only observed adverse perinatal events in mothers. The rate of perinatal complications and congenital abnormalities were comparable in each group except for the proportion of hospitalized mothers with abnormal ALT. The hospitalization rate was slightly higher in the control group (*P* = 0.016), although the ALT level in the control group was the lowest at baseline. It is possible that hepatic inflammation improved with the reduction of HBV DNA in the treated groups, while the risk of liver disease activity increased with more nutritional requirements of the third trimester of pregnancy in the control group [19, 20]. In addition, prenatal CK levels of mothers in the LdT group were higher than those in the LAM group (*P* = 0.005) and the control group (*P* < 0.001), with CK levels of one mother increasing to grade 2 (1367 U/L). Therefore, mothers taking LdT must monitor CK levels carefully to prevent myopathy [21]. Antiviral treatment did not result in a higher risk of safety problems in the newborns.

Currently, there are only limited studies focusing on the clinical outcomes during the postpartum period. In our study, most patients with treatment discontinued antiviral therapy within three months after delivery. The proportion of ALT elevation ≥ 2 × ULN in the control group was significantly higher than those in the LAM group or the LdT group within 6 months postpartum. The results demonstrated that abnormal ALT levels after delivery was not associated with LAM or LdT treatment during pregnancy and postpartum treatment cessation. Previous studies showed that increased production of adrenal corticosteroids, estrogen, and progesterone and increased amounts of regulatory T cells were related to maternal immune suppression, which prevents rejection of the fetus during pregnancy [22, 23]. Recovery in the immune system from suppression to activation after delivery resulted in hepatitis B flares [24].

In conclusion, LAM or LdT was safe for mothers and fetuses in the third trimester of pregnancy. Both could effectively inhibit HBV replication and improve the success rate of blocking MTCT. Postpartum ALT elevation in pregnant mothers with a high HBV DNA viral load was not associated with LAM or LdT discontinuation after delivery. However, mothers taking LdT should carefully monitor elevated CK levels.

## Figures and Tables

**Table 1:** Baseline clinical characteristics of pregnant women infected with hepatitis B virus among three groups.

Variable	LAM group (*n* = 363)	LdT group (*n* = 1283)	Control group (*n* = 978)	*P*
Age (years)	27.6 ± 4.1	28.0 ± 3.9	28.1 ± 4.1	0.107
Primigravida (*n*, %)	290 (79.9)	1018 (79.3)	765 (78.2)	0.732
HBeAg positive (*n*, %)	351 (96.7)	1257 (98.0)	962 (98.4)	0.159
Treatment experienced (*n*, %)	10 (2.8)	38 (3.0)	29 (3.0)	0.967
ALT (U/L)	42 (8–572)	37 (6–493)	32 (5–404)	<0.001
Rate of abnormal ALT (%)	76 (20.9)	290 (22.6)	184 (18.8)	0.090
HBV DNA (log_10_ IU/mL)	7.34 ± 0.52	7.64 ± 0.51	7.57 ± 0.57	<0.001

Values presented as mean ± SD or median (range), unless otherwise noted. ALT: alanine aminotransferase; HBeAg: hepatitis B e antigen; HBV: hepatitis B virus.

**Table 2:** Comparison of perinatal complications of pregnant mothers infected with hepatitis B virus among three groups.

Variable	LAM group (*n* = 363)	LdT group (*n* = 1283)	Control group (*n* = 978)	*P*
Premature rupture of membrane (%)	67 (18.5)	205 (16.0)	159 (16.3)	0.522
Postpartum hemorrhage (%)	17 (4.7)	96 (7.5)	57 (5.8)	0.093
Meconium staining of the amniotic fluid (III degree) (%)	29 (8.0)	89 (6.9)	84 (8.6)	0.336
Oligohydramnios (%)	17 (4.7)	36 (2.8)	26 (2.7)	0.130
Hospitalization due to abnormal liver function tests (%)	0	3 (0.2)	10 (1.0)	0.016
Fetal distress	5 (1.4)	35 (2.7)	18 (1.8)	0.185

**Table 3:** Comparisons of offspring of pregnant mothers infected with hepatitis B virus among three groups.

Variable	LAM group (*n* = 363)	LdT group (*n* = 1283)	Control group (*n* = 978)	*P*
Gestational age (weeks)	39.1 ± 1.3	38.9 ± 1.3	39.0 ± 1.4	0.088
Gender (male/female)	192/174	685/613	530/454	0.842
Infant weight (g)	3373 ± 466	3345 ± 430	3344 ± 442	0.523
Infant length (cm)	50.1 ± 1.1	50.0 ± 1.1	50.0 ± 1.7	0.624
Premature birth/low birth weight (%)	15 (4.1)	56 (4.3)	39 (4.0)	0.916
Birth abnormalities (%)	9 (2.5)	26 (2.0)	33 (3.4)	0.129
Apgar at 1 min	10 (3, 10)	10 (5, 10)	10 (4, 10)	0.081
Apgar at 5 min	10 (3, 10)	10 (7, 10)	10 (5, 10)	0.314
Apgar at 10 min	10 (3, 10)	10 (8, 10)	10 (3, 10)	0.571

Birth abnormalities include birth defects, stillbirth, and death within 24 hours of birth.

## Data Availability

The data used to support the findings of this study are available from the corresponding author upon request.
